# *Bifidobacterium breve* Promotes the Pathogenesis of IBS by Downregulating the Expression of Ferroptosis‐Related Molecule ERBB1: A Mendelian Randomization Mediation Analysis

**DOI:** 10.1155/humu/6705631

**Published:** 2026-05-20

**Authors:** Lian Mo, Yue Wu, Yan Zhou, Youcheng He, Lifeng Wei, Chunyu Zhou, Shuyu Cai, Jianye Yuan

**Affiliations:** ^1^ Clinical Research Center, Longhua Hospital, Shanghai University of Traditional Chinese Medicine, Shanghai, China, shutcm.edu.cn

**Keywords:** ferroptosis, gut microbiota, irritable bowel syndrome, Mendelian randomization

## Abstract

**Objective:**

Although gut microbiota dysbiosis has been associated with irritable bowel syndrome (IBS), the specific roles of individual bacterial strains in the pathogenesis of IBS remain incompletely elucidated. Additionally, whether these bacterial strains exert their effects through the ferroptosis pathway remains unclear. Therefore, this study is aimed at investigating the impact of ferroptosis‐related molecules regulated by gut microbiota on IBS development using Mendelian randomization (MR) mediation analysis.

**Methods:**

Genome‐wide association study (GWAS) data for gut microbiota were acquired from the FINRISK study, and protein quantitative trait loci (pQTL) data were retrieved from the deCODE database. Ferroptosis‐related genes and their downstream molecules were identified using the FerrDb V2 database; these molecules were then intersected with pQTL data to obtain ferroptosis‐related pQTLs. IBS datasets were derived from the FinnGen R12 database. A two‐step MR mediation analysis was performed, with the inverse‐variance weighted (IVW) method designated as the primary analytical approach. Eligible results were defined by three criteria: *p* value < 0.05, mediation proportion > 10%, and consistency between mediation effects and total effects. Heterogeneity analysis, pleiotropy testing, and sensitivity analysis were further conducted to verify the robustness of the results.

**Results:**

*Bifidobacterium breve* exhibited a negative correlation with v‐erb‐b2 avian erythroblastic leukemia viral oncogene Homolog 1 (ERBB1) (beta1 = −0.125). In turn, ERBB1 was negatively correlated with IBS (beta2 = −0.223), whereas *Bifidobacterium breve* showed a positive total effect on IBS (beta_all = 0.168). The mediation proportion of this pathway was 16.57% (*p* < 0.05).

**Conclusion:**

*Bifidobacterium breve* may promote the pathogenesis of IBS by downregulating ERBB1, suggesting that increased ferroptosis susceptibility in intestinal epithelial cells may play a role in this pathway. This finding provides novel insights into the underlying mechanisms of IBS and may offer a potential target for IBS intervention.

## 1. Introduction

Irritable bowel syndrome (IBS) is a common functional gastrointestinal disorder defined by chronic abdominal pain and disrupted bowel habits. With a global prevalence of approximately 11%, it imposes substantial socioeconomic burdens on healthcare systems and affected individuals worldwide [1]. The pathogenesis of IBS involves complex multifactorial interactions, including dysregulation of the brain–gut axis, visceral hypersensitivity, and low‐grade intestinal inflammation; however, the underlying molecular mechanisms driving disease development remain incompletely elucidated [2].

In recent years, the regulatory role of the gut microbiota—often referred to as the “second genome” of the human body—has attracted growing attention in IBS research. Epidemiological and experimental studies have consistently reported reduced abundance of short‐chain fatty acid (SCFA)–producing bacteria in IBS patients. These bacteria are well recognized for their ability to suppress intestinal inflammatory responses by maintaining the integrity of the gut barrier and regulating immune homeostasis, highlighting a potential link between gut dysbiosis and IBS pathogenesis [3, 4]. Despite these observations, the specific molecular pathways through which the gut microbiota modulates IBS development remain not fully characterized.

Ferroptosis, a newly identified form of programmed cell death, is driven by iron‐dependent lipid peroxidation and regulated by distinct molecular mechanisms, including inactivation of glutathione Peroxidase 4 (GPX4) and peroxidation of membrane polyunsaturated fatty acids (PUFAs) [5]. For example, nuclear factor erythroid 2‐related Factor 2 (NFE2L2) can activate GPX4 to counteract ferroptosis [6], while Cathepsin B promotes ferroptosis by translocating to the nucleus to induce DNA damage and activating STING1‐dependent autophagy pathways [7]. Accumulating evidence indicates that excessive ferroptosis impairs the function of the intestinal epithelial barrier and hinders mucosal repair [8]. In contrast, certain metabolites derived from the gut microbiota (capsiate) may facilitate intestinal tissue repair by inhibiting ferroptosis‐related pathways in the host [9]. Collectively, these findings suggest that the gut microbiota–ferroptosis axis may act as a critical bridge connecting gut dysbiosis to the pathogenesis of IBS.

Mendelian randomization (MR) is a robust analytical approach that uses genetic variants as instrumental variables to mitigate confounding biases inherent in traditional observational studies. This method has emerged as a powerful tool for dissecting complex causal pathways in biomedical research [10]. With the rapid accumulation of proteomic protein quantitative trait loci (pQTL) data, researchers are now able to apply MR mediation analysis to investigate multistep causal pathways involving exposures, mediators, and outcomes [11]. Notably, no studies to date have utilized this integrated approach to unravel the regulatory network underlying the “gut microbiota–ferroptosis‐related molecules–IBS” axis.

In the present study, we established an MR mediation analysis framework by integrating gut microbiota genome‐wide association study (GWAS) data, ferroptosis‐related pQTLs, and IBS datasets from the FinnGen R12 cohort. Through comprehensive MR analyses and mediation effect assessments, we aimed to clarify whether the gut microbiota promotes IBS pathogenesis by modulating the expression of ferroptosis‐related molecules, thereby providing novel insights into the molecular mechanisms of IBS.

## 2. Materials and Methods

### 2.1. Data Sources and Analysis

Gut microbiota GWAS data were obtained from the FINRISK study [12], which collected fecal samples from 7231 individuals for genomic sequencing. Following rigorous quality control procedures and exclusion of outliers, the final dataset included 5959 participants, with 473 gut microbial taxa identified. Instrumental variables (i.e., single‐nucleotide polymorphisms, SNPs) for these 473 taxa were filtered using the following parameters: *p* < 5 × 10^−6^, clumping window size = 10,000 kb, and linkage disequilibrium (LD) threshold *r* ^2^ < 0.1. The pQTL data were sourced from the deCODE database (https://www.decode.com/summarydata/) [13], which contained 4907 raw files. These pQTL datasets were filtered based on the following criteria: SNPs with *p* < 5 × 10^−8^, clumping window size = 10,000 kb, and *r* ^2^ < 0.1; this filtering process resulted in 4759 pQTL files. Different significance thresholds were applied according to the characteristics of the corresponding datasets and genetic instruments. For gut microbiota traits, a suggestive significance threshold (*p* < 5 × 10^−6^) was used to retain sufficient instrumental variables, given the polygenic architecture and relatively limited number of genome‐wide significant loci for microbiota features. In contrast, a genome‐wide significance threshold (*p* < 5 × 10^−8^) was applied for pQTL instruments to ensure stronger genetic associations and reduce false‐positive instruments. Ferroptosis‐related genes and their downstream molecules were identified using the FerrDb V2 database (https://www.zhounan.org/ferrdb/current/). Intersection analysis between these pQTL data and ferroptosis‐related entries yielded 165 ferroptosis‐associated pQTLs. The IBS dataset was derived from the FinnGen R12 database (https://www.finngen.fi/en/), which encompassed 407,993 participants, including 13,268 IBS cases and 394,725 healthy controls.

### 2.2. Statistical Analysis

This study adopted a two‐step MR mediation analysis to explore whether gut microbiota affects the pathogenesis of IBS by regulating ferroptosis‐related molecules. The specific analytical procedures were as follows: (1) Calculate the total effect (beta_all) of 473 gut microbial taxa on IBS, (2) compute two sets of effect values: the effect of 473 gut microbial taxa on 165 ferroptosis‐related pQTLs (beta1) and the effect of these 165 ferroptosis‐related pQTLs on IBS (beta2), and (3) calculate the indirect (mediated) effect (beta12) as the product of beta1 and beta2 and determine the direct effect (beta_dir) by subtracting *β*12 from *β*_all. The mediation proportion was further calculated using the following formula: (beta12/beta_all) × 100*%*. Finally, only results meeting the following criteria were retained: *p* value < 0.05, mediation proportion > 10%, and consistent directionality between the mediated effect and the total effect.

All two‐sample MR analyses were conducted using the “TwoSampleMR” package in R software (Version 4.4.2; https://www.r-project.org/). To reduce weak instrument bias, SNPs with an F‐statistic of < 10 were excluded from the analysis. Five MR methods were applied, including inverse‐variance weighted (IVW), weighted median (WM), MR‐Egger, simple mode, and weighted mode. Among these, the IVW method was used as the primary analytical approach due to its higher accuracy [14]. Additionally, pleiotropy was evaluated through MR‐Egger intercept tests, heterogeneity was assessed via Cochran′s *Q* tests, and sensitivity analysis was performed using leave‐one‐out analyses.

## 3. Results

### 3.1. MR Analysis of Gut Microbiota and IBS Associations

**Figure 1:**
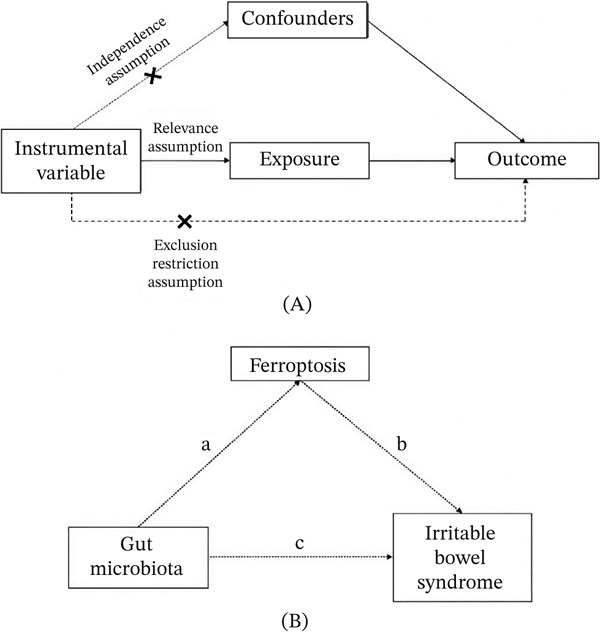
The mediation analysis framework linking gut microbiota, ferroptosis‐related molecules, and IBS. (A) MR analysis architecture. (B) Mediation analysis architecture. Pathway a: Effect of gut microbiota on ferroptosis‐related molecules (beta1). Pathway b: Effect of ferroptosis‐related molecules on IBS (beta2). Pathway c: Total effect of gut microbiota on IBS (beta_all).

**Figure 2:**
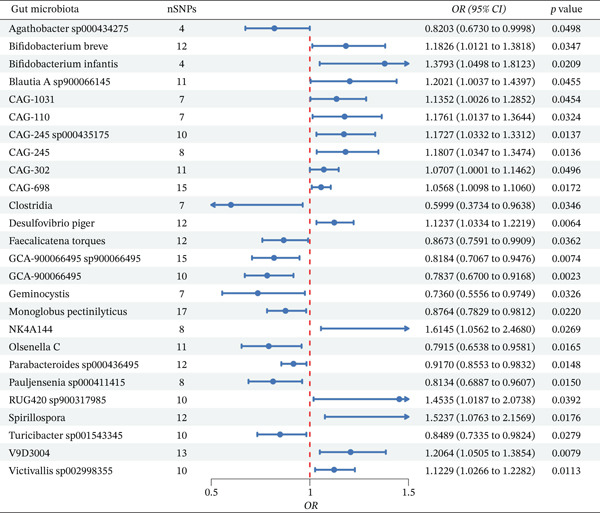
MR analysis of gut microbiota and IBS associations (IVW method). The vertical dashed line represents the null effect (OR = 1). Points to the left of the line (OR < 1) indicate potential protective effects against IBS, whereas points to the right of the line (OR > 1) indicate potential risk effects.

The mediation analysis framework that links gut microbiota, ferroptosis‐related molecules, and IBS is visually illustrated in Figure 1. The MR analysis architecture is shown in Figure 1A, and the mediation analysis architecture is shown in Figure 1B. After excluding results with pleiotropy or heterogeneity, the IVW method was applied to assess the causal effects of gut microbiota on IBS. Through this analysis, 26 gut microbial taxa significantly associated with IBS were identified (Figure 2). Among these taxa, 11 exhibited a negative correlation with IBS risk, suggesting potential protective effects against IBS. These taxa included *Agathobacter* sp000434275, Clostridia (class), *Faecalicatena torques*, *GCA-900066495* sp900066495, *GCA-900066495* (taxon), *Geminocystis* (genus), *Monoglobus pectinilyticus*, *Olsenella* C (taxon), *Parabacteroides* sp000436495, *Pauljensenia* sp000411415, and *Turicibacter* sp001543345. Conversely, 15 taxa showed a positive correlation with IBS risk, indicating their potential pathogenic roles in IBS development. These included *Bifidobacterium breve*, *Bifidobacterium infantis*, *Blautia* A sp900066145, *CAG-1031* (taxon), *CAG-110* (taxon), *CAG-245* sp000435175, *CAG-245* (taxon), *CAG-302* (taxon), *CAG-698* (taxon), *Desulfovibrio piger*, *NK4A144* (taxon), *RUG420* sp900317985, *Spirillospora* (genus), *V9D3004* (taxon), and *Victivallis* sp002998355.

### 3.2. MR Analysis of Ferroptosis‐Related Molecules and IBS Associations

**Figure 3:**
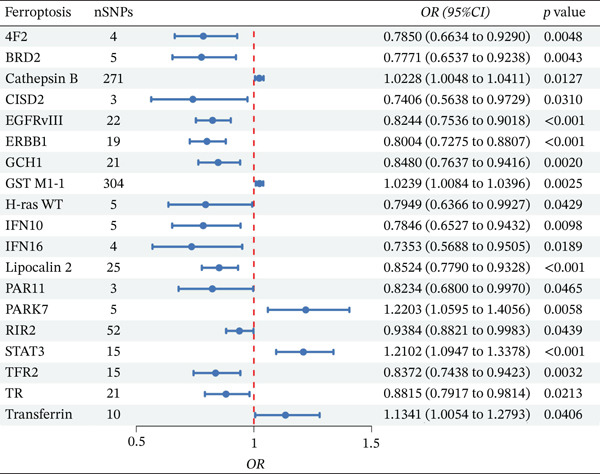
MR analysis of ferroptosis‐related molecules and IBS associations (IVW method). The vertical dashed line represents the null effect (OR = 1). Points to the left of the line (OR < 1) indicate potential protective effects against IBS, whereas points to the right of the line (OR > 1) indicate potential risk effects.

After excluding results with pleiotropy or heterogeneity, the IVW method was used to assess the causal effects of ferroptosis‐related molecules on IBS. Through this analysis, 19 ferroptosis‐related molecules significantly associated with IBS were identified (Figure 3). Among these molecules, 14 exhibited a negative correlation with IBS risk, implying potential protective effects against IBS. These molecules included BRD2, 4F2, GCH1, TFR2, PAR11, IFN10, RIR2, H‐ras WT, EGFRvIII, ERBB1, Lipocalin‐2, IFN16, TR, and CISD2. In contrast, five molecules showed a positive correlation with IBS risk, indicating their potential pathogenic roles in the development of IBS. These molecules were STAT3, GST M1‐1, PARK7, transferrin, and Cathepsin B.

### 3.3. MR Analysis of Gut Microbiota and Ferroptosis‐Related Molecule Regulatory Relationships

**Figure 4:**
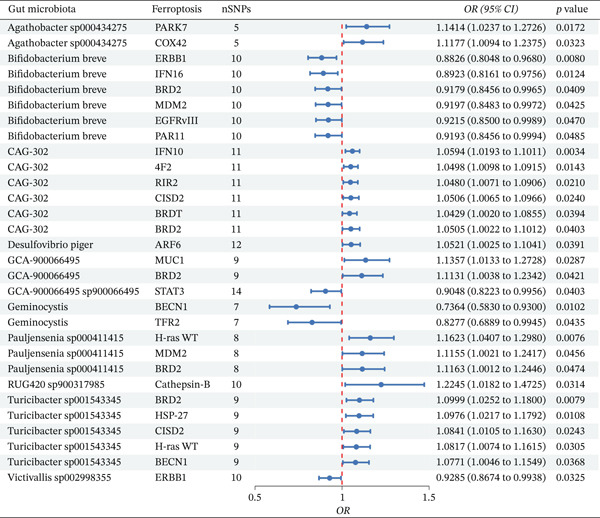
MR analysis of gut microbiota and ferroptosis‐related molecule regulatory relationships (IVW method). The vertical dashed line represents the null effect (OR = 1). Points to the left of the line (OR < 1) indicate negative regulatory associations, whereas points to the right of the line (OR > 1) indicate positive regulatory associations.

After excluding results with pleiotropy or heterogeneity, the IVW method was applied to assess the regulatory effects of gut microbiota on ferroptosis‐related molecules. Through this analysis, a total of 30 gut microbiota–ferroptosis‐related molecule regulatory pairs were identified (Figure 4), which involved 11 distinct gut microbial taxa and 21 different ferroptosis‐related molecules. The positive regulatory relationships among these pairs were as follows: *Agathobacter* sp000434275 regulated PARK7 and COX42; *CAG-302* exerted regulatory effects on IFN10, 4F2, RIR2, CISD2, BRDT, and BRD2; *Desulfovibrio piger* regulated ARF6; *GCA-900066495* affected the expression of MUC1 and BRD2; *Pauljensenia* sp000411415 regulated H‐ras WT, MDM2, and BRD2; *RUG420* sp900317985 had a regulatory role in Cathepsin B; and *Turicibacter* sp001543345 regulated BRD2, HSP‐27, CISD2, H‐ras WT, and BECN1. In contrast, the negative regulatory relationships were identified as follows: *Bifidobacterium breve* negatively regulated ERBB1, IFN16, BRD2, MDM2, EGFRvIII, and PAR11, *GCA-900066495* sp900066495 exerted negative regulatory effects on STAT3, *Geminocystis* (genus) negatively regulated BECN1 and TFR2, and *Victivallis* sp002998355 had a negative regulatory role in ERBB1.

### 3.4. MR Mediation Analysis of Gut Microbiota–Ferroptosis‐Related Molecules–IBS Pathways

Among the 30 regulatory pairs identified in Figure 4, only the *Bifidobacterium breve*–ERBB1–IBS pathway satisfied all three criteria (*p* < 0.05, mediation proportion > 10%, and consistent directionality of mediated and total effects).

By integrating the above results, the MR mediation analysis was performed to screen for valid regulatory pathways. All final validated pathways are summarized in Table 1 for comprehensive reference. Taking the specific pathway involving *Bifidobacterium breve* and ERBB1 as a representative example, the key effect values were calculated as follows: (1) The regulatory effect of *Bifidobacterium breve* on ERBB1 (beta1) was −0.125, indicating a negative regulatory relationship; (2) the causal effect of ERBB1 on IBS (beta2) was −0.223, suggesting that ERBB1 is negatively associated with IBS risk; and (3) the total causal effect of *Bifidobacterium breve* on IBS (beta_all) was 0.168, reflecting a positive association between *Bifidobacterium breve* and IBS risk. The negative beta1 indicates that *Bifidobacterium breve* downregulates ERBB1; because ERBB1 itself has a protective effect on IBS (negative beta2), its downregulation results in a pathogenic net effect, as reflected by the positive mediated effect (beta12 = beta1 × beta2 = 0.028). Thus, *Bifidobacterium breve* may promote the pathogenesis of IBS by downregulating ERBB1 expression, and this regulatory process may further increase ferroptosis susceptibility, potentially contributing to the development of IBS.

**Table 1:** MR mediation analysis of gut microbiota–ferroptosis‐related molecules–IBS pathways.

Gut microbiota	Ferroptosis	Path a (beta1)	Path b (beta2)	Total effect (beta_all)	Direct effect (beta_dir)	Indirect effect (beta12)	Proportion mediated (%) (95% CI)	*p* value
*Bifidobacterium breve*	ERBB1	−0.125	−0.223	0.168	0.140	0.028	16.57 (2.14–30.99)	0.024

To confirm the reliability of this pathway, multiple validation analyses were conducted. Heterogeneity and horizontal pleiotropy were assessed across the three MR components of the key *Bifidobacterium breve*–ERBB1–IBS mediation pathway, including *Bifidobacterium breve*–ERBB1, ERBB1‐IBS, and *Bifidobacterium breve*–IBS. Cochran′s *Q* test and the MR‐Egger intercept test showed no significant heterogeneity or horizontal pleiotropy across these three components (all *p* > 0.05; Table S1). Additionally, leave‐one‐out analysis did not indicate that the overall causal estimate was substantially driven by any single instrumental variable, further supporting the robustness of this mediation pathway.

## 4. Discussion

Through MR analysis of gut microbiota, ferroptosis‐related molecules, and IBS, this study yielded three key findings: (1) A total of 26 gut microbial taxa were identified to be associated with IBS pathogenesis, (2) 19 ferroptosis‐related molecules were found to be linked to IBS, and (3) 30 regulatory relationships existed between gut microbiota and ferroptosis‐related molecules. Furthermore, mediation analysis revealed, for the first time, that *Bifidobacterium breve* promotes IBS pathogenesis by downregulating ERBB1 (a ferroptosis‐related molecule). This finding provides critical mechanistic insights into how gut microbiota influences IBS development.

Among the 26 IBS‐associated gut microbial taxa identified in this study, the effects of most taxa are consistent with recent research. Mars et al. [15] reported reduced abundance of butyrate‐producing microbiota in IBS patients. Butyrate is known to regulate the secretory functions of the host; its deficiency may exacerbate energy metabolism dysfunction in intestinal epithelial cells, impair the intestinal barrier, and ultimately worsen IBS symptoms. Similarly, butyrate‐producing taxa identified in the present study—such as *Agathobacter* and *Faecalicatena torques*—are inferred to exert protective effects against IBS. Additionally, Clostridia (a class of bacteria) can promote bile acid synthesis and excretion while accelerating intestinal transit, which may be particularly beneficial for patients with constipation‐predominant IBS [16].

As a key component of the gut microbiota, the role of *Bifidobacterium* in maintaining intestinal homeostasis has attracted significant attention. Previous studies suggest that *Bifidobacterium* influences gut health by modulating intestinal barrier function, regulating immune responses, and producing metabolites [17]. Notably, however, *Bifidobacterium breve* in this study exhibited a promotive effect on IBS pathogenesis, which contradicts conventional understanding. Generally, *Bifidobacterium* species are thought to upregulate the expression of tight junction proteins (ZO‐1 and claudin‐1) to restore intestinal barrier integrity, elevate the levels of anti‐inflammatory cytokines (IL‐10), and reduce proinflammatory factors (IL‐8 and IL‐4)—thereby alleviating IBS symptoms [18].

The opposing effect of *Bifidobacterium breve* observed in this study may stem from strain‐specific actions or host genetic heterogeneity. For instance, Niu et al. [19] reported that *Bifidobacterium breve* strains H4‐2 and H9‐3 enhanced the expression of tight junction proteins (ZO‐1 and claudin‐1) and increased acetate/butyrate levels in a dextran sulfate sodium (DSS)–induced colitis model, ultimately improving intestinal inflammation. Intriguingly, *Bifidobacterium breve*—the first *Bifidobacterium* species isolated from infant feces [20]—is itself a butyrate‐producing bacterium. Szczuko et al. [21] observed that elevated butyrate levels in infants may promote the growth of gas‐producing bacteria, leading to abdominal discomfort; they further emphasized the need to investigate the interactions between specific taxa (*Bifidobacterium breve*) and SCFAs. These findings highlight the necessity of conducting clinical validation across different *Bifidobacterium* subtypes, including distinct strains of *Bifidobacterium breve*. Clinically, this finding does not negate the overall therapeutic potential of probiotics, but highlights the importance of strain‐specific evaluation before probiotic selection in IBS patients. Future probiotic strategies may benefit from incorporating strain‐level characterization and host genetic or molecular risk assessment to better identify individuals who may respond differently to specific probiotic strains.

Gut dysbiosis contributes to IBS pathogenesis by inducing low‐grade intestinal inflammation, damaging epithelial tight junctions, and impairing barrier function [22]. These processes further underscore the critical role of epithelial cell death in IBS development. Ferroptosis, an iron‐dependent form of programmed cell death, has been implicated in various diseases, including gastrointestinal disorders [23]. Studies have shown significantly elevated ferroptosis in the inflamed mucosal epithelial cells of patients with IBD; inhibition of ferroptosis in these cases can promote intestinal barrier repair and alleviate inflammation [24]. Although these findings do not directly confirm ferroptosis′s involvement in IBS pathogenesis, they provide indirect evidence of its potential role, and the 19 ferroptosis‐related molecules identified in this study are consistent with recent mechanistic insights. Nevertheless, the ferroptosis relevance of some identified molecules is based on database annotation and prior evidence from non‐IBS disease contexts. Therefore, their specific regulatory roles in IBS‐related ferroptosis require further experimental confirmation.

For example, phosphorylation of STAT3 can induce epithelial ferroptosis, thereby driving the development of ulcerative colitis (a type of IBD) [25]. Transferrin exacerbates ferroptotic damage by mediating iron uptake via receptor‐mediated endocytosis, leading to cellular iron overload [26]. ERBB1, a key member of the EGFR family and a critical regulator of intestinal epithelial homeostasis, plays a role in protecting the intestinal epithelial barrier [27]. Recent studies have demonstrated that aberrant EGFR signaling modulates the expression of ferroptosis‐related genes, thereby influencing cellular susceptibility to ferroptosis [28, 29]. Mechanistically, EGFR can suppress ferroptosis by inhibiting autophagy‐dependent ferritin degradation and iron release via the YAP/mTOR pathway [30]. GPX4 is a central antiferroptotic enzyme that detoxifies phospholipid hydroperoxides, and impaired GPX4 activity leads to lipid peroxide accumulation and ferroptotic cell death [5, 6]. In addition, the interaction between mTOR and GPX4 signaling has been reported to modulate autophagy‐dependent ferroptosis [31], suggesting that ERBB1/EGFR‐related YAP/mTOR signaling may intersect with the GPX4‐centered lipid peroxide detoxification system. This mechanistic link may partly explain how ERBB1 downregulation could increase ferroptosis susceptibility in intestinal epithelial cells and thereby contribute to IBS pathogenesis.

This study also identified 30 regulatory relationships between gut microbiota and ferroptosis‐related molecules. Although no direct studies have reported these specific interactions, emerging evidence highlights the role of microbial metabolites in modulating ferroptosis. For instance, secondary bile acids can activate the TFR‐ACSL4 axis to promote ferroptosis [32], 5‐hydroxytryptamine (5‐HT) and 3‐hydroxyanthranilic acid (3‐HA) can inhibit ferroptosis by scavenging free radicals [33], and histamine can accelerate ferroptosis via the STAT3‐SLC7A11 pathway [34]. Notably, butyrate—the primary metabolite of *Bifidobacterium breve*—exhibits biphasic effects on ferroptosis: Low physiological doses suppress ferroptosis by enhancing mitochondrial function, while high pathological doses promote lipid peroxidation (a key driver of ferroptosis) [35]. This bidirectional regulation further supports the notion that *Bifidobacterium breve* may drive IBS pathogenesis through the modulation of ferroptosis.

Based on GWAS data and mediation analysis, we identified for the first time that *Bifidobacterium breve* promotes IBS pathogenesis by downregulating the expression of ERBB1 (a ferroptosis‐related molecule). Mechanistically, ERBB1 may suppress ferroptosis; therefore, downregulation of ERBB1 by *Bifidobacterium breve* may increase ferroptosis susceptibility in intestinal epithelial cells, thereby contributing to IBS development.

## 5. Limitations

This study has several limitations. (1) The ERBB1 pQTL data were from whole blood (reflecting systemic expression), while its expression in intestinal tissues (epithelial/lamina propria cells) may differ [36]. This discrepancy could mismatch the estimated ERBB1 effect with its actual intestinal role in IBS, affecting mediation pathway interpretation. (2) Although *Bifidobacterium breve* promotes IBS by downregulating ERBB1, ERBB1 may influence IBS via nonferroptosis pathways (regulating epithelial proliferation/barrier repair). Experimental validation (ferroptosis‐targeted cell/animal models) is needed to confirm ferroptosis‐specific mechanisms. (3) Despite strict SNP filtering (F‐statistic ≥ 10) to reduce weak instrument bias, residual pleiotropy may remain in gut microbiota–associated SNPs. These SNPs could affect IBS via off‐target pathways, thereby biasing causal effect estimates and impacting pathway reliability [37]. (4) The genetic summary data used in this study, including gut microbiota GWAS from the FINRISK study, IBS GWAS from FinnGen, and pQTL data from deCODE, were mainly derived from European ancestry populations. Therefore, replication in non‐European cohorts is needed to confirm the generalizability of our findings. (5) The two‐step MR mediation analysis assumes approximately linear relationships among the exposure, mediator, and outcome. Potential nonlinear or threshold effects were not examined in this study and should be further investigated in future studies.

## 6. Conclusion

By integrating GWAS data and applying MR mediation analysis, this study delineated the causal effects of distinct gut microbial taxa and ferroptosis‐related molecules on IBS pathogenesis, as well as the regulatory interrelationships among these factors. Furthermore, this study suggests that *Bifidobacterium breve* may promote the pathogenesis of IBS by downregulating ERBB1, with increased ferroptosis susceptibility in intestinal epithelial cells potentially playing a role in this pathway. These findings open new avenues for therapeutic research targeting the gut microbiota–ferroptosis axis in IBS.

## Author Contributions

L.M.: conceptualization, methodology, formal analysis, investigation, and writing original draft; Y.Z., Y.W., Y.H., L.W., C.Z., and S.C.: investigation, data curation, and validation; J.Y.: conceptualization, resources, supervision, project administration, funding acquisition, and writing—review and editing.

## Funding

This work was supported by the National Natural Science Foundation of China (Grant No. 82474457).

## Disclosure

All authors consent to publish this work.

## Ethics Statement

The authors have nothing to report.

## Conflicts of Interest

The authors declare no conflicts of interest.

## Supporting information


**Supporting Information** Additional supporting information can be found online in the Supporting Information section. Table S1: Heterogeneity and horizontal pleiotropy tests for the key mediation pathway.

## Data Availability

The data are available on request from the authors.
