# Supplementary material for: Bifidobacterium breve Promotes the Pathogenesis of IBS by Downregulating the Expression of Ferroptosis‐Related Molecule ERBB1: A Mendelian Randomization Mediation Analysis
Source: Hum Mutat. 2026 May 20;2026:6705631. doi: 10.1155/humu/6705631 (PMC13189460; doi:10.1155/humu/6705631)
Supplement: Supplementary file 1 — Supporting Information Additional supporting information can be found online in the Supporting Information section. Table S1: Heterogeneity and horizontal pleiotropy tests for the key mediation pathway. [file HUMU-2026-6705631-s001.docx]

**Supplementary Table S1** Heterogeneity and horizontal pleiotropy tests for the key mediation pathway

| MR component | Cochran’s *Q* *P* value | MR-Egger intercept *P* value |
| --- | --- | --- |
| Bifidobacterium breve-ERBB1 | 0.2936 | 0.0551 |
| ERBB1-IBS | 0.8174 | 0.3981 |
| Bifidobacterium breve-IBS | 0.0738 | 0.0906 |

**Note:** Cochran’s *Q* test was used to assess heterogeneity, and the MR-Egger intercept test was used to evaluate horizontal pleiotropy. All P values were > 0.05, indicating no significant heterogeneity or horizontal pleiotropy across the three MR components.
